# Retraction: HCRP-1 regulates cell migration, invasion and angiogenesis via Src/ FAK signaling in human prostate cancer

**DOI:** 10.7150/ijbs.66407

**Published:** 2021-11-10

**Authors:** International Journal of Biological Sciences

The article (Chen F, Wu J, Teng J, Li W, Zheng J, Bai J. HCRP-1 regulates cell migration, invasion and angiogenesis via Src/ FAK signaling in human prostate cancer. *Int J Biol Sci* 2020; 16(2):342-352. doi:10.7150/ijbs.38112) has been retracted due to concerns on multiple image duplications as identified by PubPeer users. This raises concerns about the integrity of the data presented in the paper.

